# Examining the ethical and social issues of health technology design through the public appraisal of prospective scenarios: a study protocol describing a multimedia-based deliberative method

**DOI:** 10.1186/1748-5908-9-81

**Published:** 2014-06-21

**Authors:** Pascale Lehoux, Philippe Gauthier, Bryn Williams-Jones, Fiona A Miller, Jennifer R Fishman, Myriam Hivon, Patrick Vachon

**Affiliations:** 1 Department of Health Administration, University of Montreal, Institute of Public Health Research of University of Montreal (IRSPUM), Montreal, Canada; 2 School of Industrial Design, University of Montreal, Montreal, Canada; 3 Department of Social and Preventive Medicine, University of Montreal, IRSPUM, Montreal, Canada; 4 Institute of Health Policy, Management and Evaluation, University of Toronto, Toronto, Canada; 5 Biomedical Ethics Unit, Social Studies of Medicine Department, McGill University, Montreal, Canada; 6 IRSPUM, University of Montreal, Montreal, Canada

**Keywords:** Health technology, Ethics, Audiovisual based-elicitation methods, Prospective scenarios, Deliberative methods, Public involvement

## Abstract

**Background:**

The design of health technologies relies on assumptions that affect how they will be implemented, such as intended use, complexity, impact on user autonomy, and appropriateness. Those who design and implement technologies make several ethical and social assumptions on behalf of users and society more broadly, but there are very few tools to examine prospectively whether such assumptions are warranted and how the public define and appraise the desirability of health innovations. This study protocol describes a three-year study that relies on a multimedia-based prospective method to support public deliberations that will enable a critical examination of the social and ethical issues of health technology design.

**Methods:**

The first two steps of our mixed-method study were completed: relying on a literature review and the support of our multidisciplinary expert committee, we developed scenarios depicting social and technical changes that could unfold in three thematic areas within a 25-year timeframe; and for each thematic area, we created video clips to illustrate prospective technologies and short stories to describe their associated dilemmas. Using this multimedia material, we will: conduct four face-to-face deliberative workshops with members of the public (n = 40) who will later join additional participants (n = 25) through an asynchronous online forum; and analyze and integrate three data sources: observation, group deliberations, and a self-administered participant survey.

**Discussion:**

This study protocol will be of interest to those who design and assess public involvement initiatives and to those who examine the implementation of health innovations. Our premise is that using user-friendly tools in a deliberative context that foster participants’ creativity and reflexivity in pondering potential technoscientific futures will enable our team to analyze a range of normative claims, including some that may prove problematic and others that may shed light over potentially more valuable design options. This research will help fill an important knowledge gap; intervening earlier in technological development could help reduce undesirable effects and inform the design and implementation of more appropriate innovations.

## Background

In health services and policy research, it is rarely questioned whether the design of new health technologies has been well thought out, *i.e.*, the shape they take, the functions they perform, and what users may accomplish through their use [1]. Yet, once implemented into healthcare systems, certain innovation design assumptions and features such as intended use, complexity, impact on autonomy, and appropriateness may align poorly with users’ needs and expectations (clinicians and patients) and with healthcare system challenges such as sustainability, access, and equity [2,3]. While those who design and implement technologies make several social and ethical assumptions on behalf of users and society more broadly [3,4], there are very few tools to examine prospectively whether such assumptions are warranted and how the public define and appraise the desirability of health innovations. As a result, not only may questionable social transformations be fostered, but alternative designs may be ignored or abandoned early in the design process [5].

This paper describes the protocol of a three-year study whose goal is to examine the ways in which public deliberations of prospective sociotechnical scenarios—a method developed in the Netherlands [6]—enable a critical examination of the social and ethical issues underlying health technology design. In this protocol, a health innovation is understood as comprised of social and technical components that contribute to redefining states of health and illness, often involving broader and subtle transformations of the whole healthcare and social services delivery system [7]. We approach values as cultural constructs that may be more or less widely shared and so are open to interpretation and negotiation [8]. More specifically, the perceived desirability of a given innovation may refer to valuable goals (*i.e.*, social and technical outcomes that may be attained through the use of a health technology) and features that are posited as valuable in themselves (*i.e.*, properties that are considered as having social and technical appeal, such as accuracy, immediacy, reduced invasiveness, etc.) [9]. Additional definitions of the terms used in this protocol are listed below:

•‘**Usability issues**’ may arise when over-, under- or misuse of a technology affects users’ quality of life, safety, autonomy, mobility, etc.

•The term ‘**designers**’ refers to the scientists, clinicians, engineers and entrepreneurs who are fully engaged in bringing an innovation to the market.

•While recognizing that experts and non-experts possess varying levels and kinds of knowledge, following Burns *et al.*[10], the term ‘**publics**’ refers to *all* members of society.

•We use ‘**expertise**’ to refer to the ease with which someone can talk about a given innovation based on his/her experiential, scientific and/or practical knowledge [11]. This implies that a chronic disease patient could be an expert on, say, using a dialysis machine, while a radiology technician would be a non-expert with respect to this technology. Hence, individuals may move from the position of expert to that of non-expert depending on the issues under discussion.

•We use the term ‘**deliberation**’ to highlight the presence of a reasoning process that becomes explicit through a conversation between two or more individuals [12].

•The term ‘**normative assumptions**’ refers to sets of norms, principles, preferences and values that are tacitly understood, within a professional, disciplinary or cultural community, as the right way of interpreting the world in which an innovation is evolving [13].

•‘**Judgments**’ are socially situated appraisals that rely on knowledge *and* normative assumptions, summarizing the perceived desirability of an innovation (*e.g.*, highly valuable, potentially risky or to be avoided).

### Research objectives and conceptual approach

Our empirical work focuses on three thematic areas where innovations are already emerging and which will alter significantly the moral landscape of future generations [6,14]: enhancement technologies in teenagers, preventive interventions for genetically ‘at risks’ adults and ageing in a high-tech world. Applying a mixed-methods study design, our specific objectives are to:

1. analyze the ways in which members of the public, in face-to-face and online multimedia-based deliberative environments, reason and deliberate about the desirability of technical and social changes that may affect three thematic areas within a 25-year timeframe;

2. identify the usability and ethical issues raised by various design assumptions and features in these three thematic areas; and

3. assess the extent to which the sociotechnical scenario method fosters critical, reflective and creative reasoning, and deliberations regarding the design of health innovations.

In order to generate in-depth findings that can inform health innovation design and implementation, we adopt a Science and Technology Studies (STS) perspective that recognizes that technologies, as well as their designers and users, are value-laden. STS scholars argue that society and technology are necessarily co-constitutive [15], influencing each other’s evolution and setting the stage for changes to unfold in both spheres. Philosophers of technology add to this observation that morality also evolves through new sociotechnical practices [6]. For instance, abortion had to be socially and morally legitimized before prenatal screening could emerge as a viable innovation. Stem cell research would not be as widespread today but for the implementation of *in vitro* fertilization (IVF) techniques that generate ‘surplus’ embryos. While moral concerns about the status of embryos have pushed scientists to find alternative ways of producing stem cells, such concerns have also been significantly transformed by the use of IVF.

Accordingly, the STS perspective sheds light on the reciprocal relationships between values, society, and technology. This is why we choose to examine the perspectives of public since they constitute the social fabric through which innovations take shape and are implemented. Our research is also grounded in the work of Boltanski and Thévenot [16] on how judgments are formed and articulated in an attempt to settle socially shared situations. From an empirical standpoint, we adopt an empirical ethics approach that seeks to understand not only how actors themselves mobilize values, but also ‘the implicit normativity within facts and technologies’ ([17]: 70). This approach relies on social science methods to generate insights into value-laden social practices, contrasting empirical observations with theoretical frameworks [18]. Below, we summarize three bodies of knowledge that informed our study.

### Literature review

#### How the design of health innovation relies on, and embodies normative assumptions

Design is an intentional endeavour, geared at problem solving but influenced by a spectrum of interests [19]. Design practices are normative because they seek to move the world closer to a preferred state of affairs [20,21], and by doing so improve or support better practices. However, design is also indeterminate [5]; although only one design materializes, designers can foster several possible competing futures. The technology to be designed ‘is not one thing’ to all individuals involved in the process: ‘Each individual’s perspective and interests are rooted in his or her special expertise and responsibilities. Designing is a process of bringing coherence to these perspectives and interests, fixing them in the artefact’ ([21]: 187). Designers of health technology rely on various kinds of market analyses and forecasting, and their work is strongly shaped by factors that are either internal to the organization, such as strategic planning, profitability and market shares, or constitutive of the regulatory and business environment [22,23]. Those who design technologies are also informed by ‘clinical champions’ who have their own views about the relevance and fit of an innovation within clinical practice [24].^a^ The most powerful and socially legitimate claims in a technology design project usually involve efficacy, safety, and what is considered ‘good for the patient’ [25,26]. In such appraisals, medical experts’ judgments can be shaped by limited and untested social assumptions (*i.e.*, alleged costs to healthcare systems of having children be born with Down syndrome) [13]. Medical experts may define social needs and preferences in ways they think are appropriate given their patients’ expectations. Because they are themselves members of society, medical experts’ assumptions may be similar to or even the same as those of other people. Yet, their arguments are often considered authoritative by the public, patients, and policymakers, so their assumptions need to be unpacked and complemented by other perspectives before innovations become established [4].

As more complex forms of health intervention are continually emerging, scholars have continued to voice arguments in favour of including the public in discussing the putative benefits and risks of such technologies [27]. While public engagement often occurs ‘downstream’, as technologies are entering the market, it may also happen ‘upstream’ and early on in the development process [8]. Some authors suggest that patients and/or non-experts should participate in health innovation policy to ensure that policies are ‘based’ on science yet ‘informed’ by so-called social values [28]. This view tends to assume that scientific facts form a ‘hard’ indisputable core around which ‘soft’ preferences are attached [29]. This view also explains the linear, sequential processes that are often proposed for public involvement: processes that tend to start from science in order to end up with normative decisions. In our study, we adopt a different stance, one that recognizes that both experts’ and non-experts’ reason on the basis of what they know and of what they value at a given point in time [30].^b^ In other words, when individuals make judgments about the desirability of a given health innovation, they are inextricably combining both normative assumptions and knowledge about the innovation.

### How prospective sociotechnical scenarios may foster reflexivity and creativity

Taking stock of the prospective methods that have been proposed since the 1960s, a Dutch team [6,31] developed an ethics research method building on sociotechnical scenarios [32]. The goal is to support reflective deliberations about technological change that may take shape in the ‘more distant future’ (10 to 30 years). Scenarios usually take a narrative form, providing a plausible, intelligible story about a putative future. Scenarios may pursue different purposes (research or policymaking), are constructed through different processes (relying on informal versus formal knowledge) and possess different characteristics (simple/few variables versus complex/interconnected events). For Boenink, ‘scenarios are descriptions of possible futures’ that are ‘historically informed speculations’ which seek to foster the self-reflexivity of participants ([6]: 6)^c^.

Given our own focus on technology design, we expect prospective scenarios to also spark creativity: participants may reframe or emphasize desirable/undesirable goals and features that have hitherto been marginal in the context of current healthcare systems [33]. Davies and Powell [34], who reviewed alternative approaches to knowledge transfer and exchange—such as storytelling, the arts, or immersive learning—argue that the use of fiction draws attention to the significance of ‘language and metaphor in shaping how ideas are shared and received’ and may help social research better draw in experience, emotion, and appeals to identity. Such elements are pivotal to the ways in which individuals express deeply held beliefs [35]. Narratives ‘permit the exploration of difficult issues in a non-threatening form’, they ‘help individuals and groups address the affective dimensions of being’ and they ‘open up multiple perspectives’ that can lead to concrete changes ([34]: 6–7). For instance, adopting a structured process of facilitated discussions called ‘experience-based design’ in which clinical staff and patients shared their stories and experiences, Bate and Robert [36] showed how it enabled solving quality and safety issues by having staff and patients co-design particular healthcare services. Our study thus posits that prospective scenarios enable individuals to envision and relate to historically informed potential futures, possibly fostering through an immersion in fiction their creativity and reflexivity about the practical and moral implications of technological and social developments in healthcare.

### How deliberative processes help make judgments explicit

While research on public involvement is steadily growing, one assumption is that participants may choose to revise their position if they are presented with new evidence or arguments [37]. Without deliberation, however, knowledge cannot circulate, challenge normative assumptions, and eventually transform judgments [38]. Because one becomes aware of taken for granted moral norms when they ‘are no longer able to provide satisfactory responses to new problems’ ([39]: 5), our prospective scenarios will expose participants to usability issues (*e.g.*, how over-, under- or misuse of technology may affect safety, autonomy, mobility, etc.) and normative challenges to which they are likely to relate both affectively and rationally.

By examining a two-week long online forum hosted for the residents of the New York City area on how to rebuild the former World Trade Center, Black [40] showed how ‘dialogic moments’ that enable open-endedness and human connection helped explore more fully the complexities of participants’ commitments and values. For Black, dialogue occurs through interaction that triggers ‘momentary experiences of profound mutual awareness of the other person’ ([40]: 95). Such moments can address conflicts based on moral difference, since participants may realize how they are tied to one another and always part of a larger group. When participants are made accountable to other actors [16], dialogue may change their views: ‘Each speaker incorporates and reinterprets the other’s contributions in his or her own way […] the process of trying to convince others may alter one’s own mode of expression but also the reasons one finds convincing’ ([12]: 58). Further, Stromer-Galley and Muhlberger ([41]: 187), in surveying 179 residents of Pittsburgh who deliberated online about solutions to the problem of underused schools, found that ‘expressions of disagreement do not generally harm [participants’] evaluations’ of deliberative processes. They thus recommend that public deliberations ‘experiment with enforced devil’s advocacy’ since it ‘may be useful for more fully exploring all sides of an issue’. Similarly, Walmsley [33], who examined a citizen panel whose task was to design a biobank, underscored how ‘persistent disagreements’ among participants pushed them to be more creative in order to find solutions that could eventually reconcile their varying principles. Overall, when discussing prospective scenarios and considering unsuspected moral challenges, productive group disagreements and dialogical moments may help disclose, clarify, and challenge participants’ normative assumptions and judgments.

## Methods

**Figure 1:**
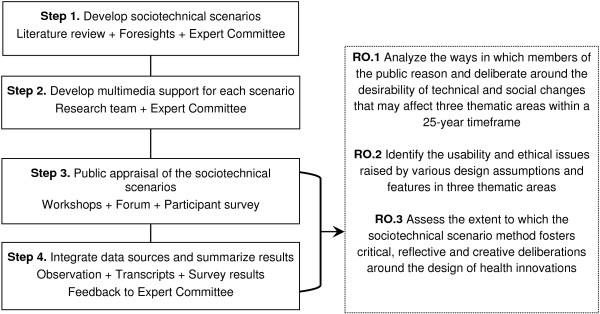
**Research steps and research objectives (RO).** Note: Steps one and two were completed in January 2014. Steps 3 and 4 should be finalized by the end of 2015.

We adopt a mixed-method study design in which the principles of qualitative research predominate [42]. Figure 1 offers a schematic representation of the four steps underlying our study design, which involves creating three video clips and six short stories, hosting four deliberative workshops and facilitating an online forum.

### Step one: developing prospective scenarios in three thematic areas

We chose to structure our prospective scenarios around three thematic areas (not specific diseases or technologies), not only to maximize the ability of participants to identify with the stories being told, but also to enable setting the putative technical and social changes within a broad moral landscape. Together, the thematic areas help address a large spectrum of usability and ethical issues, while each emphasizes the needs and preferences of different social groups across the life course: the use of enhancement technologies in teenagers; preventive interventions for genetically ‘at risk’ adults; and ageing in a high-tech world.

These thematic areas were chosen by considering the requirements for conducting a solid and instructive comparative empirical ethics analysis, including relevance, diversity, coherence, and feasibility [42]. First, these themes address topics that are already capturing the attention of policymakers and attentive publics because the real-life implications of such developments may have to be dealt with in their lifetime. Second, the themes offer enough diversity to explore the subtleties, prejudices and nuances by which participants may ponder normative issues in different contexts, for different human beings (*i.e.*, increasing teenagers’ performance versus offsetting elderly people’s frailty). Third, the common thread behind these themes, which will increase the coherence of our comparative analyses, is that they all address the technological redefinition of ‘normal’ cognitive and physical states and processes, and the growing emphasis on the ability to exercise agency over one’s body. Finally, examining three themes will provide a more solid contribution to knowledge, while keeping the empirical work and the participants’ tasks reasonable.As indicated in Figure 1, throughout our study, we will receive feedback from a committee comprised of members with expertise in: family medicine, engineering, nursing, pediatric psychiatry, bioethics, geriatric care, genetics, and public engagement (see Acknowledgments). So far, the committee members have helped us narrow down the thematic areas and have commented on drafts scenarios (Step one), they pre-tested our online forum, providing advice regarding its structure and content (Step two), and will also criticize our preliminary findings so we can develop more solid empirical analyses (Step 4).

Following the three-step method of Boenink *et al.*^d^, we first performed a literature review to document the current moral landscape for each of the thematic areas, gathering quantitative and qualitative published research on ethical aspects of health technology [43]. We paid attention to broad categories of values, such as ‘terminal values (goals or objectives), procedural values (means and process for achieving the goal), or substantive values (criteria justifying decisions and actions for goal achievement)’ ([44]: 61). The observations from this literature informed the kinds of personal and societal dilemmas that could be illustrated in our prospective scenarios.

Second, for each thematic area, we identified how plausible technoscientific developments may interact with the moral landscape and pose regulatory or policy challenges. Here, our team turned to another type of documentation that guided our reflection for choosing the technologies to be featured in each scenario. The Health Canada Science and Technology Foresight Workshop [45] identified close to thirty trends and drivers in five domains (social, technology, economy, ethics/values, environment), several of which were relevant to our thematic areas. Also, the UK Sigma Scan Horizon Scanning initiative [46], which compiled 2,000 sources and interviewed 300 experts, ‘created a cross-sectoral information basis for all foresight activities’ ([47]: 2). Sigma Scan is managed by Foresight, which reports directly to the British Government’s Chief Scientific Adviser and the Cabinet Office. There are more than 270 policy briefs, each rated in terms of its ‘impact’, ‘likelihood’, and ‘controversy’. A preliminary search had identified 51 relevant briefs classified under the following categories: healthcare (6), values (7), body and mind sciences (25), and nanotechnologies (13). These briefs helped our team to identify not only emerging technologies but also convergence across domains that various scientific developments enable (*i.e.*, neurosciences, nanomaterials, epigenetics, renewable energy, robotics, big data analysis, and information technology).

Third, integrating the literature review with these already developed foresight briefs, our research team iteratively drafted and refined prospective scenarios (an initial four-page document was created for each thematic area). From a technological standpoint, our aim was to generate scenarios that were somewhat audacious and controversial, yet empirically plausible [47]. From a sociological standpoint, our goal was to develop compelling and engaging scenarios that would draw participants’ attention to the concrete ways in which society, morality and technology influence each other. To facilitate establishing resonance with a large audience, we chose to create short stories, since ‘part of what makes stories powerful is that they are able to display values and worldviews that are typically not talked about explicitly’ ([40]: 105). A story usually pivots around the ‘quest’ of its key protagonist, which forms the ‘story theme’ [48]. Stories are made up of ‘a series of temporally ordered clauses that provide the orientation, complicating action, and resolution of the story’, while fully formed narratives may also include ‘a coda, which signifies the end of the story’ ([40]: 100). Aligned with the deliberative aim of our online forum, we chose to invite participants to develop and share their own coda.

Table 1 offers a summary of the technologies we have ‘invented’ for developing a coherent and manageable but open-ended representation of each thematic area and their corresponding story themes. For each thematic area, we devised a collective dilemma taking place in 2030 and a personal dilemma arising ten years later. Both bring forward conflicts and normative issues, but the personal dilemma focuses on the specific quests and challenges of one main character (*e.g.*, a teenager, a young adult, and an elderly person). Designed to generate comments by participants in the online forum, each text depicting a dilemma is around 500 words (*e.g.*, a format adapted for on-screen reading). Table 2 summarizes ‘tips for writing a good story’ [48] that guided our writing process. For achieving balance between the benefits and risks illustrated in the scenarios, we used the feedback of our expert committee and the patterns of ethical argumentation described by Swierstra and Rip [39], which are summarized in Table 3. These patterns pinpointed various positions that protagonists in our stories could defend.

**Table 1:** An overview of the three technologies and their associated story theme

	**Enhancement technologies in teenagers**	**Preventive interventions for genetically ‘at risk’ adults**	**Ageing in a high-tech world**
**Technologies**	A shirt with embedded sensors that provide real-time feedback about the mental state and cognitive performance of the person wearing it.	Implantable cardiac ‘rectifier’ that destroys cells genetically susceptible to cause arrhythmia later.	An assistive personal robot connected to the Internet, which can interact with individuals and the built environment (using face, voice and object recognition).
	Used with meditation techniques, the shirt can help one learn about oneself	The rectifier transmits data to a centralized system where experts confirm its plan of action	The robot is used at home and can ‘learn’ from its owner by asking questions and memorizing responses
**Story theme**	Finding ways to be oneself while building skills and competence to move towards adulthood.	Pondering genetic risks and uncertainties while deciding whether or not to intervene in an otherwise healthy body.	Adapting to successive transitional states and a gradual loss of mental and physical abilities while seeking to remain autonomous as long as possible.

**Table 2:** **Tips for writing good stories (adapted from**[48]**)**^**e**^

	
**Theme**	‘A theme is something important the story tries to tell us—something that might help us in our own lives.’
	Sheppard recommends not getting ‘too preachy’ and ‘not to say what the moral is.’ It is more interesting for readers if the theme grows out of the story with subtlety and nuances, so they feel they have learned something for, and by themselves.
**Plot**	The plot usually revolves around a ‘conflict or struggle that the main character goes through.’ It can invoke personal needs and feelings, involve another character, or be in response to ‘the way things are.’ The protagonists ‘should win or lose at least partly on their own, and not just be rescued by someone or something else.’
	In a good story, by trying to solve things that are problematic, the characters learn and grow. This is what the ‘story theme’ is about.
	Sheppard underlines that ‘a novel can have several conflicts, but a short story should have only one.’ This conflict should grow in intensity, reach a ‘climax’ and then resume.
**Characters**	Sheppard recommends laying out and defining the characters—who they are, how they think, what they do—before starting to write. Knowing those characters well will bring coherence to the story plot. These characters should have some traits that readers relate to, or even make them care about them.
	Sheppard also stresses that ‘a main character should have at least one flaw or weakness. Perfect characters are not very interesting. […] And they don’t have anything to learn.’ Similarly, a ‘bad guy’ should also possess some positive qualities.
**Setting**	Sheppard recommends setting the story ‘in a place and time that will be interesting or familiar.’
	In our case, since the scenarios are prospective, we describe settings that depart from those in which healthcare is currently delivered and from those in which people are now interacting. Nevertheless, our scenarios have to be plausible and therefore settings that are sufficiently concrete and puzzling are selected.

**Table 3:** **A summary of patterns of ethical argumentation (adapted from**[39]**)**^**f**^

**Consequences (hoped for)**	
*Enthusiasts*	*Sceptics*
Promises (*e.g.*, increased control over the world and increased well-being)	Plausibility (uncertainty)
	Adverse side effects (cost/benefit)
	Can the good not be produced otherwise (*e.g.*, search for alternative)
	Is the envisioned good really a good
Unforeseen problems will be solved by future solutions	
**Rights and principles (tensions between the individual and the collective)**	
Positive right to the technology (*e.g.*, people should have access to the technology)	Principle is wrong
	Principle is null in another culture/setting
	Principle is right in the abstract, but does not apply to the issue Principle is right, but it supports the opposite conclusion, or it conflicts with another one that is more pressing
Negative right to the technology (*e.g.*, free to acquire it as long as it does not harm others)	
**Justice (distributing the costs and benefits)**	
Different bases: Equality; Merit; Need; Chance	
Through trickle down effects, technology will benefit the whole society	Without political intervention, those in need or who are economically disenfranchised will never benefit
**Good life**	
Humankind should move forward/upward	Knowing when/where to stop (‘not to play God’)
	Respecting natural limits (not create ‘monsters’)
	Preserving humanness and pushing it to flourish ‘as-it-is’
	Social problems cannot be solved by technical fixes
	Technology cannot be controlled
Frontiers/limits can be transgressed	
Promethean vision	
**Relationship between technology and morality**	
Deterministic (technology’s internal logic)	Voluntarists (technology is socially malleable)
External forces too strong (markets, economies, scientific competition)	Technology is steerable in a morally desirable direction
	Pessimists (technology as a moral problem)
	Technology is already immoral as it is
	Technology will manoeuvre us (‘slippery slope’)
Optimists (technology as a moral solution)	
Precedent (not novel moral issues)	
Society will habituate itself	

### Step two: developing multimedia support for each thematic area

The use of audiovisual material is a particularly effective means for enabling informed deliberations among participants and to help articulate their perspectives, because it facilitates the grasping of technoscientific principles with which they are not familiar and increases their ‘reactivity’ by putting ‘images at work’ ([49]: 866). According to Evans and Plows [11], when a specific topic is under consideration, members of the public may have no expertise, interactional expertise (able to converse intelligently about a given topic), or contributory expertise (able to contribute knowledge to a given field). When developing our scenarios, we assumed that participants’ expertise may fall into any of these three categories. As such, the technical and clinical issues were described assuming that participants did not hold expertise.

By choosing to develop video clips, we put forward an ‘audiovisual, elicitation-based’ data collection strategy [49] that aims to enrich the data to be gathered without, however, imposing a pre-determined reading of the issues at play. When using audiovisual-based elicitation methodologies, participants are asked to respond to what they have seen/heard, to comment or contest the expressed claims, and to explain whether or not it alters their understanding of the issues. The video clips were developed with both the face-to-face workshops and the online forum in mind. We created for each thematic area a three-minute video clip, using a combination of static and dynamic images, including actors and voice-over narrative. To guide the pre-production, production, and post-production stages of the video clips, we used a set of tools (scripts, storyboards, sequence-by-sequence shooting plan) that we had applied in a previous project and described elsewhere [50]. The aim of each video clip was to describe the prospective technologies—providing answers to questions such as ‘how does it work’ and ‘what does it do’—and to illustrate the context in which the technologies would be used by 2040.

### Step three: face-to-face and online deliberations

While knowledge on Internet-based research is still growing [51], we found one study that compared face-to-face and online focus groups with participants affected by colon cancer. Campbell *et al.* ([52]: 100) observed that while similar themes emerged from both, online groups may attract participants who ‘would be physically unable to attend a face-to-face focus group’ and those ‘who feel reluctant to discuss sensitive health issues in person’. The authors note, however, that the need to type responses may lead participants to shorten or omit certain comments, an issue that might have been exacerbated by their chosen synchronous format. Black [40] argues that storytelling is likely to be fostered in asynchronous online posting because participants can take the time to respond without interruption, express their ideas or tell their stories more completely than if they were in a face-to-face interaction. Thus, the added value of gathering data through two types of deliberative environment is that it increases participant diversity (because of varying time/space constraints) and it enables the examination of variations in how normative claims are articulated and shared (oral, immediate, and public versus written, delayed, and anonymous).

### Recruitment strategies

While these two environments call for distinct data collection strategies, an integrated recruitment strategy will be deployed for both. We adhere to the definition of Burns *et al.* ([10]: 184) for whom ‘the simplest and most useful definition of the public is every person in society’.^g^ We will foster the participation of individuals from all walks of life; the only exclusion criteria we intend to apply is for persons who are fully trained healthcare professionals. Multiple recruitment tools and strategies will thus be deployed in parallel by reaching out to local organizations that cater to three age groups: young adults (18 to 25); adults (30 to 55); and people over 60 years old. The goal will be to reach participants who may share an interest in social and technology issues, but who may do so from a large range of normative perspectives and reasoning processes. For instance, we will target groups that organize reading clubs, conferences, cultural events, or training activities (for young entrepreneurs, occupational-based networking, or retired people). To ascertain the interest and relevance of each organization serving as an intermediary, a study recruiter (a communication expert who will also act as the workshop/forum moderator) will reach out to them by phone or in person meeting. Then, an electronic invitation letter will be circulated through various newsletters, websites, and social media. The invitation will provide the coordinates of our workshop/forum moderator, our study website and information regarding the Health Research Ethics Board approval. Through a phone conversation, the moderator will gather basic demographic and socioeconomic information about each potential participant. From the pool of interested participants, four groups of 10 members will be assembled using a reasoned sampling technique [42]. We will aim at forming one group of young adults, one group of adults, one group of people over 60 years old, and one mixed group. The goal will be to obtain sufficient homogeneity in socioeconomic status to ensure that the exchanges are comfortable and lively, yet diversified [53]. Those who are not available at the day and time set for the workshop will be invited to participate in the online forum. Akin to a snowballing strategy, participants will be asked to share the invitation to the forum with friends and relatives.

### Deliberative workshops

Morgan [54] argues that the ability to observe the extent and nature of participants’ agreement and disagreement is a unique strength of focus group research. Although methodologists generally advocate seeking homogeneity in group composition around age, gender or occupational status, deliberative focus groups bring together participants who can engage in more challenging discussions [53]. Hollander [55] underscores that the context-dependent data generated through group discussions is socially situated. This perspective emphasizes the larger social structures within which discussions take place, as well as the various motivations and expectations of participants and the position(s) they represent as they contribute to ongoing discussions [56]. The group moderator is pivotal in facilitating open and detailed exchange with and among participants. Crossley even points out that a moderator may deliberately try to trigger a strong reaction, in order to obtain ‘valuable insights into how people may actually respond’ to health interventions ([57]: 1479).

To support an in-depth discussion of our three prospective technologies, we will host four 3.5-hour deliberative workshops. Once each participant has introduced her- or himself, the video related to the first thematic area will be shown and then each participant will be asked to share with the group two or three features of the technology he/she sees as desirable as well as two or three undesirable features. A group discussion will ensue focusing on potential ways to improve the technology. The same structure will be applied to the other two thematic areas, with about one hour per theme and a 15-minute break (refreshments and snacks will be served). Forty-five Canadian dollars will be given to each participant at the end of the workshop in recognition of their participation.

### Asynchronous online forum

Our forum will be hosted on a login/password-secured page and facilitated by the same moderator as the workshops. Defining a fixed timeframe in asynchronous online forums creates momentum, fostering a more intense participation. Our forum will thus run over a four-week period, starting after the fourth workshop will have taken place. Participants will be invited to read and agree to a consent form, to view a brief animation explaining the study, to read the dilemmas, to view the videos and to respond to open-ended questions to kick-start a collective debate. Participants will be able to return to the forum whenever they wish and comment on each other’s comments. A cheque of forty-five Canadian dollars will be sent by regular mail to each participant at the end of the forum.

### Step four: integrating the data sources and summarizing the results

Our analyses will rely on three main data sources: observation; deliberative content; and a self-administered participant survey. Table 4 indicates how this mixed-method strategy capitalizes on the respective strengths of the datasets that will be generated. While the deliberative content represents a rich source of data for documenting the process and outcome variables related to all three research objectives (ROs), observation provides additional insights into the processes underlying RO.1 and RO.3. The survey, by documenting the views of all participants, including those who may occupy the less ‘discursive place’ in a group, will add nuance to our qualitative findings and support a structured assessment of the prospective method (RO.3).

**Table 4:** Data sources

**Research objectives**	**Variables of interest**	**OBS**	**DEL**	**SURV**
**RO.1** To analyze how members of the public, in face-to-face and online environments, reason and deliberate around the desirability of sociotechnical changes in three thematic areas.	**Processes:**			
	Ways in which participants reason, agree/disagree, and ponder the desirability of sociotechnical changes within/across thematic areas.	√	√	√
	The influence of group deliberations over the formation of one’s judgments (including the group moderator).	√	√	√
	**Outcomes:**			
	Similarities/differences within/across thematic areas in participants’ knowledge claims, normative assumptions and argumentative patterns.		√	√
	Similarities/differences between the two deliberative environments in how views are articulated and shared.		√	√
**RO.2** To identify usability and ethical issues raised by various design assumptions and features in three areas	**Outcomes (to be interpreted in light of the scholarly literature):**			
	Usability and ethical issues that are addressed/ignored by participants within/across thematic areas.		√	
	Design assumptions and features considered desirable/undesirable, that predominate, are reframed or ignored by participants.		√	
**RO.3** To assess whether the sociotechnical scenario method fosters critical, reflective and creative deliberations around the design of health innovations	**Processes:**			
	Appraisal of the audiovisual and written components of each scenario.	√	√	√
	Participants’ level of engagement throughout the process and ability to relate to the protagonists’ and other participants’ stories.	√	√	√
	Expressions of creativity, reflexivity and critical sharing of information.	√	√	√
	**Outcomes:**			
	Ways in which participants envision and describe the value of sociotechnical developments in healthcare.		√	√
	Critical observations toward design assumptions and features, and scope/depth of proposed alternatives (participants’ own conclusions).		√	√

OBS: observation; DEL: face-to-face and online deliberations; SURV: survey.

### Observation

The workshops and online forum will be moderated by someone with experience in group processes and directly observed by someone trained in qualitative research. The observer will take notes and the discussion will be recorded and transcribed verbatim (4 × 3.5 hrs = 16 hrs of audiotaped discussion). Our observation technique is ‘non-participant’ because the observer plays no instrumental role in the group discussion, although the observer has a certain influence simply by being present [58]. By contrast, the moderator is a participant-observer because this person plays a pivotal and overt role in the workshop discussions and is active in the online forum. From a qualitative research standpoint, this represents an empirical opportunity to examine more broadly the context in which the data collection process unfolds and its influence on the phenomenon [56,58].

For the workshops, detailed observation notes will be recorded in two steps. First, during the event, the observer will record short notes on a pre-structured form describing the characteristics of the interactions between participants (*e.g.*, climate, turn taking, flow/intensity of interactions). Second, the day after the workshop, an annotated summary of the deliberations will be written, integrating and structuring the notes recorded on the form, and exploring analytical insights. Observations of the online forum dynamics will be complemented by navigation data. For instance, the number of page visits per participant, the number of comments per participant and the ratings of the comments will be downloaded into a spreadsheet. Using a Wordpress platform, we will follow the Journal of Medical Internet Research checklist, which covers methodological and technical issues, including ethics (*i.e.*, consent, privacy, security) and calculation of ‘response’ rates (*e.g.*, the view, participation and completion rates of unique visitors, based on the use of cookies and log file analysis) [59].

### Content of deliberations

Verbatim transcripts of the workshops and the content of the online forum will be integrated into the qualitative indexing software QDA Miner. They will be analyzed using a mixed coding strategy, involving a set of predetermined codes based on the list of variables described in Table 4 and a set of empirically-induced codes. For the workshops and the online forum, both intra- and inter-group analyses will be performed (see below).

### Self-administered participant survey

All participants will be asked to complete an online survey when the forum closes. Table 5 lists the open- and close-ended items of the survey. Given that our recruitment strategy emphasizes diversity, and as qualitative research standards of rigour suggest, the survey will also enable the gathering of demographic and socioeconomic data, contributing to an in-depth description of our participants [60]. A specific set of items to compare the two deliberative environments is reserved for participants who will have attended one workshop. With the survey data and given the number of respondents (limited to 40 in the workshops and expected to be around 65 in the forum), descriptive statistics will be compiled (distribution of frequencies, measures of central tendency). To explore whether different patterns of responses are observed in socioeconomic sub-groups or across thematic areas, analysis of variance (ANOVA) will be performed.

**Table 5:** The participant survey (translated from French)

**Item**	**Measurement**
**First part-Multimedia content and scenarios**	
The videos have helped me understand the technologies	5-level Likert-type scale
The videos have helped me understand the online scenarios	5-level Likert-type scale
The following video made me react the most	List of the 3 videos and ‘none’
Do you have comments about the videos?	Free text box
The online scenarios helped me reflect about questions raised by the technologies	5-level Likert-type scale
The online scenarios stimulated discussion	5-level Likert-type scale
The online scenarios that made me reflect the most are those associated with…	List of the 3 scenarios and ‘none’
I felt concerned by the dilemmas faced by the characters (Nathan, Mathis and Catherine)	5-level Likert-type scale
I felt much more concerned by the dilemma of …	List of the 3 characters and ‘none’
**Workshop and online deliberations**	
Throughout this project:	
I was brought to look at technologies differently	5-level Likert-type scale
I could consider new viewpoints	5-level Likert-type scale
I discovered effects of technology that I had never imagined	5-level Likert-type scale
I reflected more about the pros and cons of technologies	5-level Likert-type scale
I looked for additional information on the topics discussed	5-level Likert-type scale
I shared my reflections with people around me	5-level Likert-type scale
I had ideas to improve technologies around me	5-level Likert-type scale
If yes, share one idea …	Free text box
I consider that I know more about:	
The way technologies may transform society	5-level Likert-type scale
The way technologies may transform values	5-level Likert-type scale
The way individuals and society may intervene in technology	5-level Likert-type scale
The way values may influence technology design and use	5-level Likert-type scale
**Second part-Deliberative environments**	
During the workshop:	For workshop participants
I was confortable sharing my ideas	5-level Likert-type scale
I could express disagreements	5-level Likert-type scale
I voluntarily omitted expressing certain viewpoints	5-level Likert-type scale
I shared opinions that I would not have formulated as easily in writing	5-level Likert-type scale
During the online forum:	
I was comfortable sharing my ideas	5-level Likert-type scale
I could express disagreements	5-level Likert-type scale
I voluntarily omitted expressing certain viewpoints	5-level Likert-type scale
I shared opinions that I would not have formulated as easily verbally	5-level Likert-type scale
Did you prefer one of the two deliberative environments?	For workshop participants who may answer ‘none’
Why?	Free text box
**General impressions about my participation**	
Throughout the project:	
I have had the opportunity to express myself freely	5-level Likert-type scale
I was attentive to the views of other participants	5-level Likert-type scale
My arguments were well thought out	5-level Likert-type scale
I contributed to further the reflections of other participants	5-level Likert-type scale
Participating in the process required considerable efforts	5-level Likert-type scale
I remained interested throughout the experience	5-level Likert-type scale
**General impressions about the participation of other participants**	
Throughout the project:	
Participants have had the possibility to express themselves freely	5-level Likert-type scale
The arguments of the other participants appeared well thought out	5-level Likert-type scale
I have heard viewpoints that differed from mine	5-level Likert-type scale
Certain viewpoints conflicted with my values	5-level Likert-type scale
My viewpoint was well received by the other participants	5-level Likert-type scale
Group exchanges have furthered my reflections	5-level Likert-type scale
The moderator contributed to stimulate the group’s reflections	5-level Likert-type scale
The moderator respected the opinions of participants	5-level Likert-type scale
Do you have something to add about this experience?	Free text box
**Third part—Information about the participants**	
To which age group do you belong?	18 to 29; 30 to 39; 40 to 49; 50 to 59; 60 to 69; Over 70
Gender	M or F
Do you live:	By yourself? With a partner? With your family? With roommates or in a residence?
Do you self-identify with an ethnocultural community?	If yes, which one(s)?
What language(s) do you speak at home?	Free text box
Do you self-identify with one of the following religions?	Buddhism; Christianism; Hinduism; Islamism; Judaism; Other; None
Do you frequently interact with children or teenagers? (*e.g.*, many times a month)	Yes; No
Do you frequently interact with people over 65? (*e.g.*, many times a month)	Yes; No
What is your current occupation or the job you have held in the past and you most identify with?	Free text box
Which is the highest level of education you completed?	High school; Collegial; Undergraduate studies; Master’s level; PhD
How often are you in contact with healthcare services, for you or your relatives?	Never; Rarely; Occasionally; Frequently
How do you characterize your ease with technology in general?	Uncomfortable; Mostly uncomfortable; Mostly comfortable; Very comfortable
What is your household income? (that of your parents if you live with them)	Less than $20,000; $20,000 to $39,999; $40,000 to $59,999; $60,000 to $79,999; $80,000 to $ 99,999; Over $100,000

Note: The 5-level Likert-like scale was: Totally agree; Agree; Neither agree nor disagree; Disagree; Totally disagree. The possibility to answer ‘Don’t know/Doesn’t apply’ was provided.

### Data integration and analyses

Multiple data studies require a careful management of data collection and analytic processes in order to maximize the cross-fertilization of data sources throughout the study [61]. To integrate the qualitative and quantitative findings, we will use analytical tools suggested by Miles and Huberman [62], including comparative matrixes to identify patterns based on the similarities/differences observed across thematic areas, deliberative environments, participant characteristics, and chronologies, which are particularly helpful to examine conversational flows and argumentative ‘turning points’. During such exploration of the empirical material, constant comparative analysis forces one to search for empirical observations that may refute one’s emerging interpretation [63].

An inductive approach to the interpretation of our findings will prevail since the principles of qualitative research predominate in our mixed-method study design [42]. These analyses will be guided by our research objectives. For RO.1, to analyze the knowledge and normative assumptions from which participants appraise and reach ‘situated arrangements’ [16] about the value of sociotechnical changes across the three thematic areas, we will use a template that we developed for focus group research [56]. This analytical template brings forward how epistemological content and group processes influence each other, taking the moderator’s influence into account. Similarities and differences between the groups and the two deliberative environments will be examined systematically through comparative matrixes. To identify usability and ethical issues (RO.2) as required for an empirical ethics analysis, our attention will focus not only on the participants’ views, but also on the issues that are omitted or reframed. Here, comparisons across thematic areas will also be emphasized, using the scholarly literature and our expert committee to strengthen our analyses. Finally, assessing whether the participants’ reflexivity, creativity, and critical thinking have been fostered by the prospective method (RO.3) requires identifying whether and how the processes (including videos and short stories) may have supported participants’ ability to envision, describe, and make critical observations about design assumptions and features, and articulate their own alternative visions of the future.

We will host a debriefing session with the expert committee at step four once preliminary results are available. The goal will be to consolidate our analyses by having these distal collaborators ask new questions and suggest competing explanations in order to avoid the phenomenon of ‘collective persuasion’ and to identify neglected dimensions. Overall, we will apply recognized strategies for ensuring the rigour of qualitative research [60]. First, seeking to increase the credibility of our findings (a qualitative criterion similar to internal validity) and using the guideline developed by Datta [61], we will iteratively revise and adapt our study design when needed and pre-test the workshop/forum guide and survey. Second, we will provide as much information as possible regarding the participants’ characteristics and the context in which the deliberations unfold. This, coupled with the debriefing session on preliminary results with the expert committee, will contribute to defining which aspects of our findings may be transferable to other settings. Third, we will make explicit how our own assumptions regarding design features may influence data collection and analyses. Fourth, we will keep a detailed fieldwork diary, support our analyses with ‘raw’ empirical material, and facilitate access to our instruments and database (procedural accountability).

### Study status

While steps one and two were completed in January 2014, steps three and four should be completed by the end of 2015. We expect the transcription of the 16 hours of workshop deliberations, the downloading of the online comments and navigation data, the cleaning of the exit survey data, and data analysis and integration to take up to 18 months.

## Discussion

We believe this study protocol should be of interest to those who design and assess public engagement initiatives and to those who examine the implementation of innovations in healthcare. According to Giacomini *et al.* ([44]: 66), ‘clarifying the values that underlie policy options is crucial in pluralist societies facing complex questions about limited health resources, the direction of technological innovation, responses to health threats, and the goals of the healthcare system’. In fact, intervening earlier in technological development could help reduce undesirable effects and pave the way for the design and implementation of more valuable innovations [7].

The premise of our study is that preserving the sustainability of healthcare systems and implementing wisely new technologies will require solid and ‘actionable’ analyses of how certain kinds of innovations may bring a more valuable response to healthcare systems challenges and societal priorities when compared to others. One way of moving toward the development of such analyses is to understand how various stakeholders envision, value, and justify different types of innovation. Within this perspective, prospective ethical analyses may be anchored in formal, expert disciplinary knowledge and explicitly seek to provide guidance for the dissemination of innovations [3]. This type of analysis is particularly helpful for discussing policy options with other experts, decision-makers and policymakers.

By creating user-friendly tools enabling publics to ponder potential technoscientific futures, we have different but complementary goals in mind: methods and tools enabling citizens to actively contribute to contemporary debates about innovation in healthcare are needed. Seeking to overcome our limited ability to research the publics’ views is particularly important because innovation stakeholders tend to call upon a putative social ‘demand’ for technology in many different ways. If experts and policymakers are to make informed choices regarding new technologies in the best interests of society, then they need to have access to a nuanced understanding not only of the ‘lay public’s’ explicit values and beliefs, but also of their own often implicit values. The use of fiction within a deliberative context that fosters participants’ creativity and reflexivity will enable our team to examine, through a solid, detailed comparative analysis, a diverse set of normative claims, including some that may prove problematic from an ethical perspective and others that may shed light on potentially more valuable options for health innovation design. Such scholarly work will thus contribute to fill an important knowledge gap.

The limitations of our study are linked to its scope and innovative features, including the development of compelling scenarios and the management of an online forum. While philosophers of technology have accomplished important work shedding light over the profound dynamics by which human life and worldviews are transformed by technology [64], most analyses of health innovation tend to focus on a single case or area of medical practice. Although examining three thematic areas might be seen as ambitious, we feel that high-quality scholarly work can also benefit from adopting a ‘generalist’s’ perspective, which is key to our Knowledge Transfer and Exchange (KTE) philosophy. Given our experience with the prior development of public-oriented KTE tools and events (Scientific Cafés, Hinnovic blog, TEDx on Design for Health), our team acquired useful communication and technical multimedia skills and thus felt well prepared to take up the challenges of the current project.

## Endnotes

^a^For example, during the early development of medical imaging devices [24], physicians tended to favour innovations that were closely aligned with their quest for autonomy and control over expertise and tasks.

^b^Examining the ethical principles guiding the decisions of pregnant women who were offered prenatal testing in the Netherlands, Garcia *et al*. [30] found that normative preferences shaped not only the women’s decisions, but also what they already knew and had learned about the tests. When justifying their decisions, both knowledge and normativity went hand-in-hand. Hence, one needs to pay attention to how individuals’ reasoning is anchored in normative assumptions (what makes a given innovation desirable?) *and* knowledge regarding the plausibility of the innovation’s effects (are they likely to be realized?) [29].

^c^Boenink and her colleagues developed their method not only to foster public debates (it was applied in the 2010 Dutch public debate on nanotechnology: http://www.nanopodium.nl), but also with a clear scholarly goal in mind. Currently available tools to anticipate the social impacts of emerging innovations have an important epistemological drawback: “they hardly acknowledge the mutual interaction between technology and morality” ([6]: 2). In the Dutch debate, vignettes and video clips were used to illustrate various ethical and social issues and foster dialogue between citizens. Similarly, a consortium led by ethicists, social scientists and computer programmers at the Norwegian Centre for the Study of the Sciences and the Humanities at the University of Bergen launched the Technolife project (http://www.technolife.no), which relied on a combination of audiovisual and online tools. For this group, improved regulation of technoscientific developments can only begin “if we can better communicate among a greater number of concerned groups” because the ways in which these groups imagine possible futures are pivotal “to the development of technologies and the social roles they will come to play” (from their website last accessed on April 16^th^, 2014).

^d^To describe the three-step framework, Boenink [14] uses an illustration that explores the impact of molecular medicine on moral practices concerning medical experiments with human beings. Once a topic has been delineated, the first step documents and analyzes the current moral landscape. The main task is to summarize current beliefs, practices and regulations, and identify whether and how value conflicts have been settled in the recent past. The second step involves examining how a given technological development may interact with this landscape, identifying what controversies may arise [31]. The third step requires identifying, from historical and sociological analyses, what kind of closure may happen; this involves sorting out the different views and counter-views, arguments and counter-arguments that could be put forward by key stakeholders. The goal is to reduce the number of arguments to those that may be the most likely and powerful. A prospective scenario may be broken down into “phases” that help focus the participants’ attention on specific changes in morality, society and technology. In their illustration, Boenink describes four phases, each of which brings forward specific moral dilemmas that are triggered by: biomarkers and data mining (2010–2012); point of care applications (2013–2017); wet sensors and data warehouses (2018–2023); and theranostics (2024–2030).

^e^Aaron Sheppard is a children’s author who maintains web resources [48] for teachers and storytellers. Because his straightforward approach to storytelling is suited to the general audience our scenarios are shared with, his tips have helped our team develop engaging stories.

^f^According to Swierstra and Rip [39], there are argumentative patterns that structure how controversial technoscientific matters are debated. These patterns may be operating at different levels, but tend to be broad oppositions, arguments and counter-arguments.

^g^From a science communication perspective, these authors identify several overlapping groups that have varying interests and levels of knowledge concerning scientific issues, such as “mediators” (i.e., communicators, journalists, educators), decision-makers, community and interest groups, the “attentive” publics who are already interested in and reasonably well-informed about scientific activities, and publics who are not necessarily well informed [10].

## Competing interests

The authors declare that they have no competing interests.

## Authors’ contributions

All authors revised the content of the manuscript and have approved the final version. More specifically, PL is the principal investigator of the study; she is accountable for all aspects of the work, including the original idea behind the study and its development. PG, BWJ, FAM, and JRF have contributed to the study design by critically appraising preliminary versions of the research proposal before submission to the Canadian Institutes of Health Research (CIHR) in August, 2011. MH and PV have contributed in important ways to the design of the multimedia content and data collection tools (scenarios, videos, online forum and survey) while BWJ and PG participated in the pre-testing of the online forum and meetings with the Expert Committee.
